# Supplementary material for: Fresh versus Frozen Embryo Transfer in In Vitro Fertilization/Intracytoplasmic Sperm Injection Cycles: A Systematic Review and Meta-Analysis of Neonatal Outcomes
Source: Medicina (Kaunas). 2024 Aug 22;60(8):1373. doi: 10.3390/medicina60081373 (PMC11356234; doi:10.3390/medicina60081373)
Supplement: Supplementary file 1 [file medicina-60-01373-s001.zip › medicina-3142641-supplementary.pdf]

# Assessing publication bias using funnel plots and testing asymmetry with Egger's and Begg's Tests

**Figure S1. PREMATURE BIRTH**

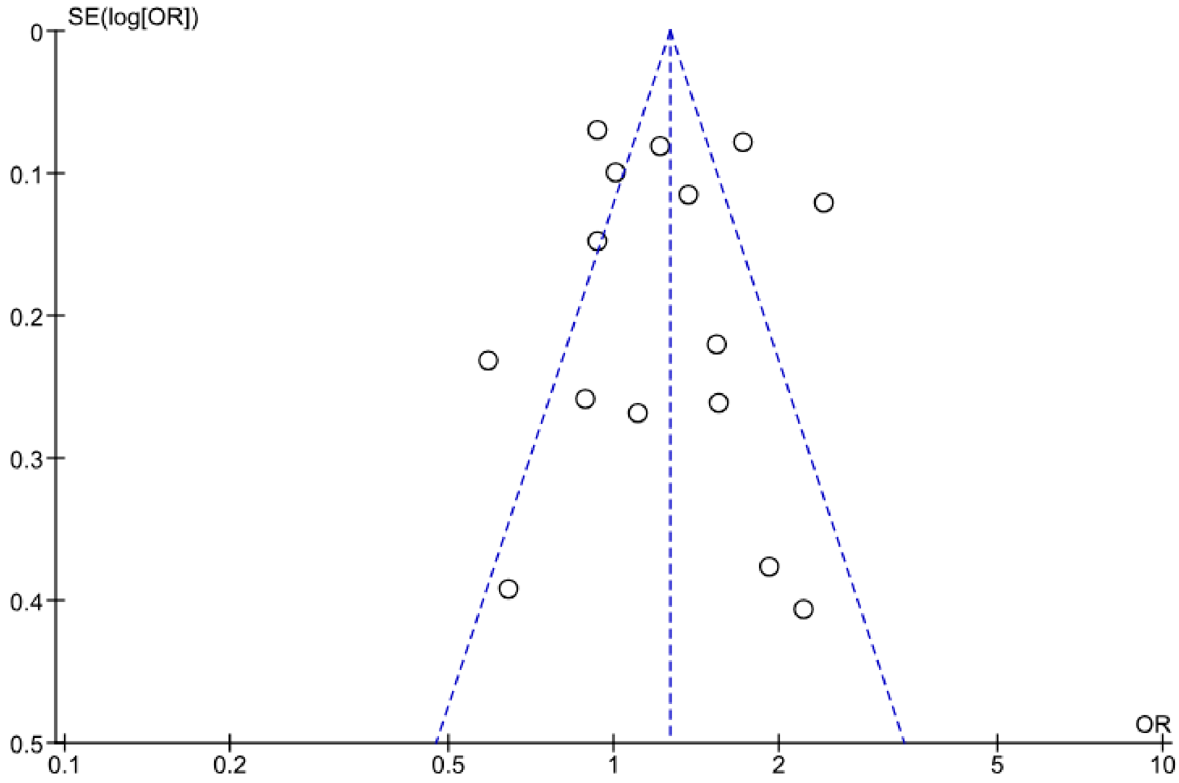


**Table S1**. *Rank correlation test for Funnel plot asymmetry*

| | Kendall's τ | p |
|---|---|---|
| Rank test | 0.200 | 0.328 |

**Table S2.** *Regression test for Funnel plot asymmetry ("Egger's test")*

| | z | p |
|---|---|---|
| sei | 0.751 | 0.453 |

**Figure S2. LBW**

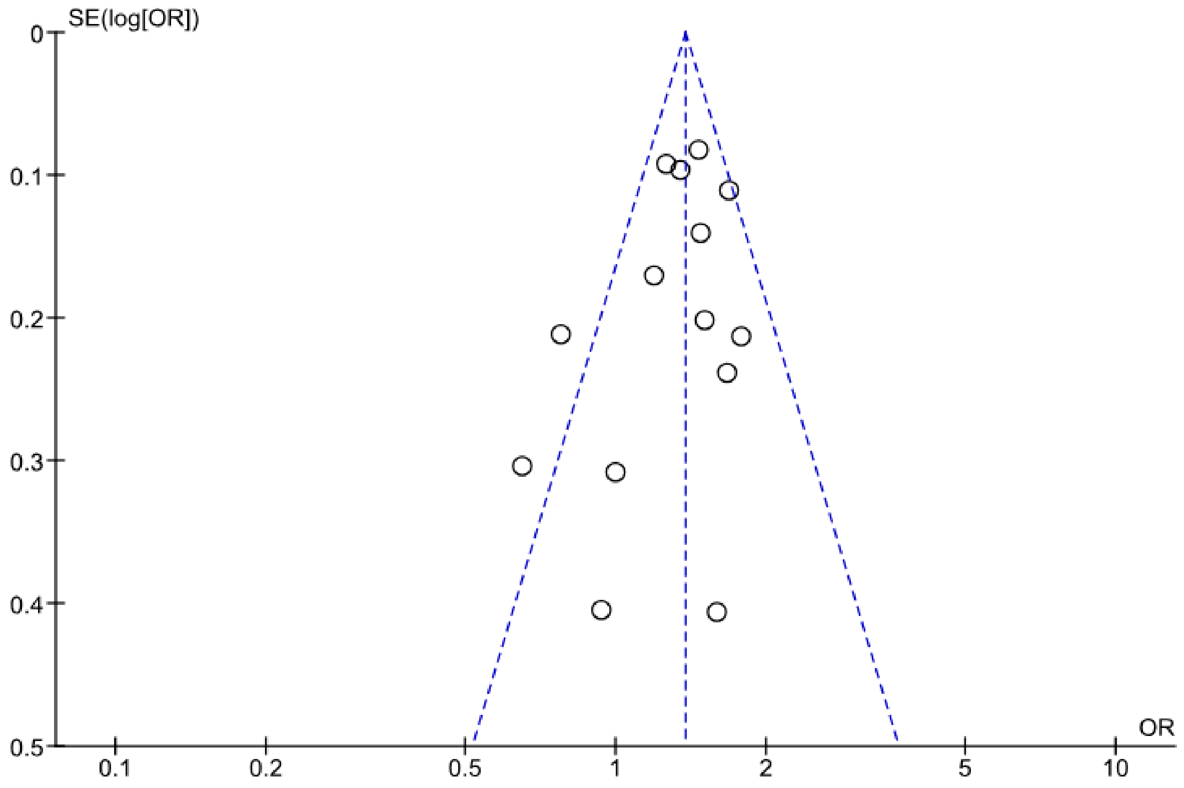


**Table S3**. *Rank correlation test for Funnel plot asymmetry*

| | Kendall's τ | p |
|---|---|---|
| Rank test | -0.121 | 0.591 |

**Table S4.** *Regression test for Funnel plot asymmetry ("Egger's test")*

| | z | p |
|---|---|---|
| sei | -1.577 | 0.115 |

**Figure S3. MACROSOMIA**

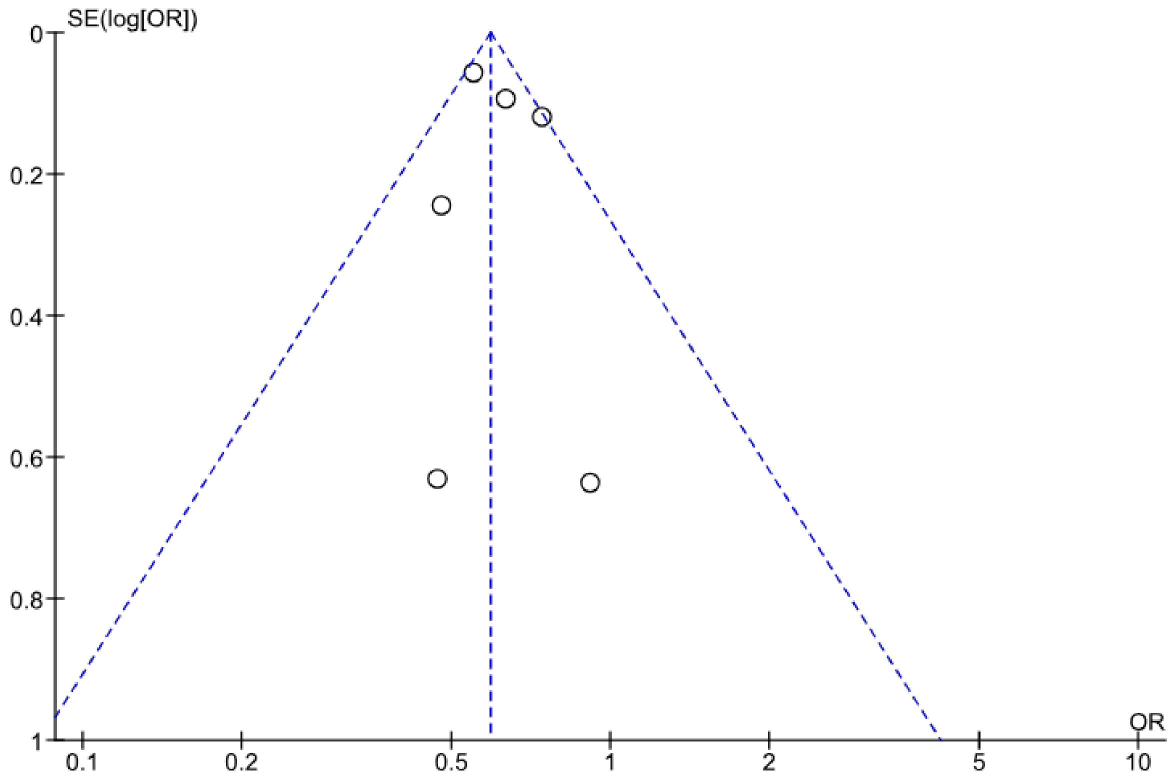


**Table S5.** *Rank correlation test for Funnel plot asymmetry*

| | Kendall's τ | p |
|---|---|---|
| Rank test | 0.333 | 0.469 |

*Regression test for Funnel plot asymmetry ("Egger's test")*

| | z | p |
|---|---|---|
| sei | 0.488 | 0.626 |

**Figure S4. SGA**

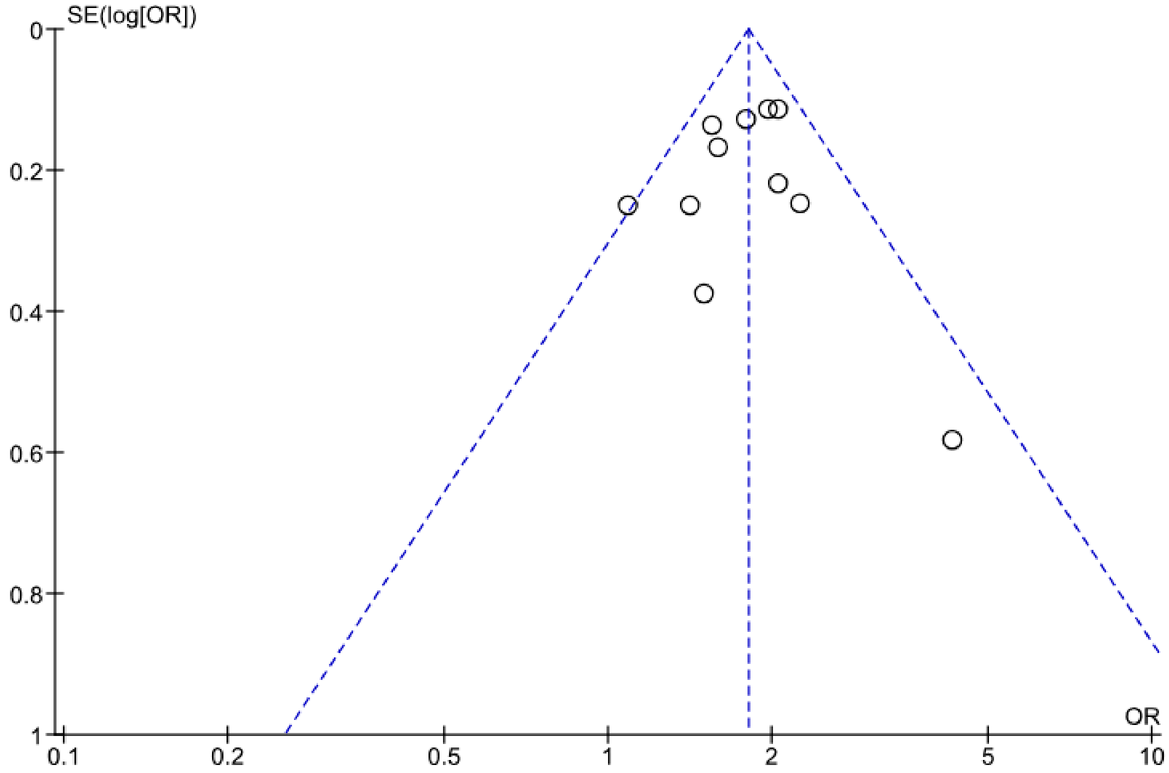


**Table S6.** *Rank correlation test for Funnel plot asymmetry*

| | Kendall's τ | p |
|---|---|---|
| Rank test | -0.127 | 0.648 |

*Regression test for Funnel plot asymmetry ("Egger's test")*

| | z | p |
|---|---|---|
| sei | 0.645 | 0.519 |

**Figure S5. LGA**

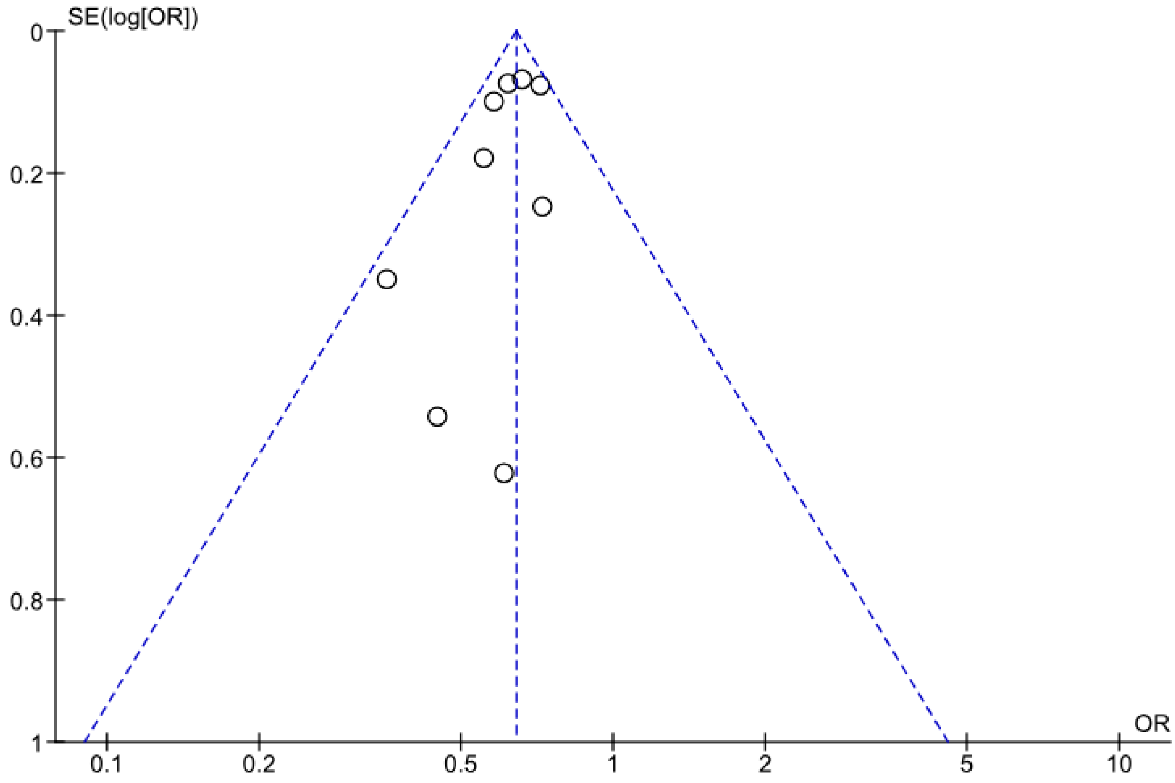


**Table S7.** *Rank correlation test for Funnel plot asymmetry*

| | Kendall's τ | p |
|---|---|---|
| Rank test | -0.167 | 0.612 |

*Regression test for Funnel plot asymmetry ("Egger's test")*

| | z | p |
|---|---|---|
| sei | -0.734 | 0.463 |

**Figure S6. CONGENITAL MALFORMATIONS**

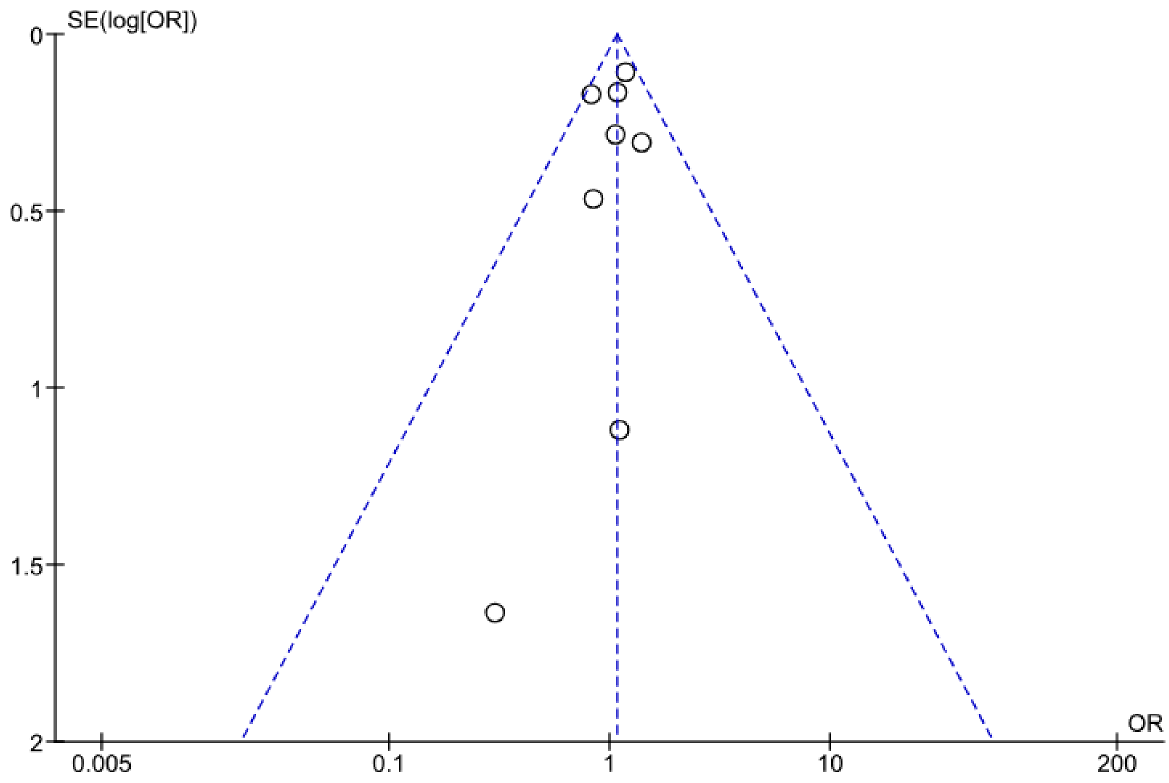


**Table S8.** *Rank correlation test for Funnel plot asymmetry*

| | Kendall's τ | p |
|---|---|---|
| Rank test | -0.143 | 0.773 |

*Regression test for Funnel plot asymmetry ("Egger's test")*

| | z | p |
|---|---|---|
| sei | -0.294 | 0.769 |
